# Urinary Incontinence in Older Adults Takes Collaborative Nursing Efforts to Improve

**DOI:** 10.7759/cureus.9161

**Published:** 2020-07-12

**Authors:** Crislyn McDaniel, Iqbal Ratnani, Saher Fatima, Muhammad Hasan Abid, Salim Surani

**Affiliations:** 1 Internal Medicine, HMS Healthcare, Houston, USA; 2 Critical Care Medicine, Debakey Heart and Vascular Center, Houston, USA; 3 Internal Medicine, Houston Methodist, Houston, USA; 4 Internal Medicine, Institute for Healthcare Improvement, Boston, USA; 5 Internal Medicine, Corpus Christi Medical Center, Corpus Christi, USA; 6 Internal Medicine, University of North Texas, Dallas, USA

**Keywords:** urinary incontinence, continence assessment, continence education, older adults, geriatrics, nursing homes, long-term care

## Abstract

There is a misconception that urinary incontinence (UI) in older adults, usually above the age of 65 is a part of aging. More than 50% of residents in long-term care (LTC) settings are affected by UI and it is associated in many cases with markedly reduced quality of life. It has become evident that incontinence can be cured or successfully managed. However, many nurses lack sufficient knowledge to intervene appropriately. The purpose of this review is to share how the collaborative efforts of nurses at all levels may lead to increased assessment and interventions of UI in this population.

## Introduction and background

Introduction

Urinary incontinence (UI) is defined as the complaint of any involuntary leakage of urine [1]. UI is a common problem in patients, especially those who are elderly, mostly above age 65, and living in nursing homes. The literature suggests that more than 70% of nursing home residents in the United States experience UI [2]. It is expected that UI will continue to be a significant problem in elderly and institutionalized persons, and will increase as the population of the United States continues to age [3-4]. Although the prevalence of UI increases with age, it should not be considered a normal part of the aging process [5]. Despite this highly prevalent condition, basic knowledge about UI and its management is lacking among nursing home staff [6]. This lack of knowledge leads to nurses not instituting measures necessary to assess and initiate treatment in these vulnerable residents [7]. Instead, UI has been alternatively viewed as an intractable problem requiring containment. A multidisciplinary team effort comprising a nurse continence advisor (NCA), a pelvic floor physiotherapist, a geriatrician, and other health professionals is needed to manage incontinence problems in the geriatric patient population [8]. Advanced practice nurses are in a unique position to provide healthcare services (i.e., training) to this group of healthcare professionals to avert the adverse consequences of untreated UI and decrease associated morbidity of the residents affected. It is a well-known fact that nursing homes in the United States are highly regulated; however, current recommendations and guidelines mandated by the federal government for UI evaluation and treatment have fallen short, as mechanisms already in place have not resulted in the desired level of care [9]. UI is often described as one of the most onerous and difficult aspects of the nursing staff’s job, resulting in poor evaluation and treatment regimens [9]. Thus, telling nursing homes that they need to change is insufficient in itself. Improving UI care practices in nursing homes requires a combination of education and cheerleading. 

Background

Urinary incontinence is extremely common, unacknowledged, and under-treated in the elderly population. Although the nurses can play a pivotal role in assessment and management this plan of care is not easily implemented and generally, the nurses in nursing homes are not performing assessments of residents with UI owing to identified environmental barriers, such as work overload and lower prioritization of UI [10]; rather concentration is on containment of urine, by use of absorbent products, without determining confounding variables [11]. Lekan-Rutledge and Collin reported assessment is often short-changed in terms of scope and depth, and that additional education and training on UI is needed [12]. Thompson also states that, while many specific tests and treatment regimens are available, evidence exists that they are underused [13]. The authors reported it is unfortunate that most facilities do not have adequately trained personnel or resources to complete the training.

One study by Watson et al. [9] found that residents with UI are subject to the inadequacy of care and do not receive the standard level of healthcare services indicated for evaluation and treatment as recommended by the Agency for Healthcare Policy and Research (AHCPR) and the Center for Medicare and Medicaid Services (CMS) guidelines. In the Watson et al. study, researchers found that the rate of UI in a nonrandom sample of 52 nursing homes was “manageable,” but assessment and treatment were rare when compared with the 1996 published recommendations from the AHCPR. Care was only given in about 20% of cases. Of the number of residents assessed, 2% of incontinent women had a pelvic exam, 15% had a digital rectal examination, and only 3% received treatment. 

Additionally, Schnelle et al. found that many nursing homes lacked documentation of the residents who were assessed, and there was no documentation of documented toileting plans. Instead, they found 99% of incontinent residents wore absorbent products. While this type of assessment is rarely documented [14], it is a sound standard of practice [15]. Thus, from a quality improvement standpoint, nursing at all levels plays a vital role in assuring appropriate care and ensuring strategies be employed when providing care to nursing home residents with UI [16].

## Review

Significance of urinary incontinence

Urinary incontinenceUI is an expensive problem for nursing homes, with an estimated annual cost of more than five billion dollars [17]. This estimated cost includes labor, laundry, and supplies [4]. Besides the financial ramifications, UI increases the risk for physical problems such as skin breakdown, for example, perineal dermatitis, skin maceration, and pressure ulcers [18]. Residents who are incontinent are also at risk for developing urinary tract infections (UTIs) that not only exacerbate incontinence but also represent a major source of sepsis in the elderly. Incontinent elderly persons are more likely to fall, either because of the sense of urgency to reach the toilet or because of slipping on a floor wet with urine [19]. Falls frequently precipitate a fracture, which could lead to immobilization and indirectly increase the episodes of incontinence. In addition, UI can cause various emotional problems, such as embarrassment, frustration, depression, and loss of self-esteem leading to social isolation, loss of independence, and institutionalization [20]. The physical and emotional problems can result in significant morbidity in terms of impaired quality of life for these vulnerable older adults. Therefore, educating nursing home staff regarding UI is a healthcare measure that will reduce the financial burden presented to nursing facilities and improve residents’ quality of life.

Review of the literature 

Nursing home staff have a lack of knowledge, training, and skills needed to provide appropriate care to patients [21]. There is very little training on incontinence designed for nurses in educational curricula, in RN staff education, and in continuing education programs. Education has been recommended, and given its strengths and relatively few weaknesses, educational programs are a feasible and effective strategy to diffuse evidence-based and best-care practice, thus translating research into nursing home practice [22]. 

Current Guidelines

The comprehensive set of guidelines for the evaluation and treatment of adult UI was first published in 1992 and further updated in 1996 by the AHCPR [23]. The current guidelines consist of a 154-page document that addresses nonspecific UI and is too complex for practice. The recommendations focus on: (1) basic evaluation of the UI, such as history; (2) physical examination and testing (e.g., a urinalysis, urine for culture and sensitivity, and post-void residual test); (3) evaluation and treatment of reversible causes of the UI; (4) further specialized testing by a urologist if warranted; and (5) treatment of the UI based on the presumed type of incontinence without further testing if conditions do not preclude. Although somewhat dated, this document continues to provide basic recommendations for the evaluation and management of UI in older adults.

Importance of Assessment

Assessment of incontinence is the key component to effective bladder restoration and must not be taken lightly. Nurses should consider the side-effects of medications and other transient causes such as excessive urination related to diabetes, UTI, or limited mobility preventing timely access to a toilet that predisposes nursing home residents to UI [24]. Identification of the underlying condition is essential prior to treatment. Once the resident’s condition is assessed, a plan of care can be developed to optimize the bladder function. Blanket plans for all residents are ineffective. Each plan must be specific to the type of incontinence. The core of any rehabilitation effort is the assessment process and only once this is completed, appropriate care is determined [25].

Supporting Evidence for Educational Intervention

Numerous educational interventions have been tested. In a systemic review, teaching healthcare staff strategies for the prevention and management of UI increased the knowledge of continence management [26]. Education programs change the attitudes and behavior of staff [1]. Similarly, Trad et al. demonstrated that nursing education and formal assessment can improve nursing compliance and management of patients with either urinary or fecal incontinence in an acute care setting [27]. Collette et al. and Rahman et al. provided empirical evidence to support an educational program as one way to increase knowledge and positively influence the behavior of staff charged with ensuring appropriate evaluation of residents with UI [21, 28]. Other authors sought to identify the factors that facilitate or diminish care providers' propensity to improve continence care in long-term care (LTC) settings [29].

Conducting a cross-sectional qualitative study using focus group methodology, the authors evaluated: staff perception of UI knowledge, UI management practices, and what factors enabled or hindered continence care in their institution. Content analysis was used. Facilitating and inhibiting elements of three individual/internal factors (beliefs about UI, attitudes towards the elderly, and knowledge about UI) emerged as important determinants of these care providers' a propensity to manage UI.

For a program to be successful, motivate the facilities and staff, have in place an efficient training process, provide good oversight, continuously monitor the process, and identify “program champions” who will take ownership of the program [29]. Therefore, educating nursing home staff about evidence-based strategies for evaluation and treatment of UI is a social investment that can reduce the burden to residents and those present in healthcare systems simultaneously. 

In another study, an important barrier in the implementation of effective incontinence treatments is the level of knowledge of the nurses concerning the assessment and treatment of UI [30]. Therefore, it is important to assess current nurses’ knowledge and practice in UI care so that nurses can receive adequate training and education. Interestingly, nurses correctly answered 73% of questions about knowledge but only performed continence-related practice about half the time [31]. Saxer et al. invited subjects to voluntarily participate in personalized instructions. After completion of the project, each participant completed a questionnaire regarding knowledge and practice. Knowledge of registered nurses increased to 96%-98% on the following three items: (1) UI can occur more often in sneezing, coughing, and walking; (2) approximately 85% of the nurses did not know the right answer to the question, admission to a home more women are incontinent than men; and (3) more than 50% of all residents in nursing homes suffer from UI. They also found that 40% of the nurses reported “never” to the documentation of UI, but 92% reported “always” to helping residents to the toilet if residents express a wish. The results of this study demonstrated nurses’ knowledge and practice about UI are in need of re-education, especially in the documentation as the responsibility for documentation lies with nurses. The authors state, “good documentation is important because this helps give each resident appropriate care”. The authors allude that this should serve as an impetus for on-the-job training, for care planning, and assessment is necessary. 

Any program proposed should ensure that educational needs are met. The educational program should consist of a series of workshops targeted not only to professional staff (RNs and LVNs) and personal care assistants but also, to operators of the facilities. The educational programs were followed by onsite consultation and guidance in dealing with specific resident situations. Hence the need for an advanced practice nurse as a consultant. The use of advanced practice nurses for instituting a successful continence program is present throughout the literature [3].

 In another randomized controlled trial carried out in 12 Australian nursing homes, it was concluded that the introduction of guideline-compliant management strategies into the nursing practice can facilitate the likelihood of evidence-based interventions for the conservative management of UI [32]. In another study, Kincade et al. used an education consultation approach for the implementation of a bladder management program. Given the high prevalence of cognitive impairment, few licensed staff, and low staff-to-resident ratios, many behavior techniques, and toileting programs are inappropriate. This study targeted community-dwellers, particularly in adult care homes. In these community settings, many of the same physical and mental impairments were shared by older adults, with similar low staff-to-resident ratios, when compared to nursing homes. The authors provided steps to develop a bladder management program that highlights effective educational and behavioral techniques that are shown to be successful. The steps within this program were to: (1) select a heterogeneous group; (2) project staff education by learning their facilities and organization, including resources and review of strategies for effective management of bladder control; (3) develop and present educational workshops utilizing a two-tier approach for the facility and resident levels (the facility-level approach focused on changes that could be made in ways of delivering care, while the resident level focused on assessment and management of bladder control problems); (4) assist in the implementation of the bladder management program by frequent facility visits providing summaries of accomplishments and planning future direction and; (5) revisions of programs based on outcomes visits and assessment of further education needs or the need for different strategies such as bulletin boards, resource notebooks, and pamphlets [33].

Competency-Based and Case-Based Approaches

Urinary incontinence is a serious clinical problem that is rarely evaluated or treated properly. A competency-based approach and case method approach encourages everyone to actively participate and use the group as a learning tool and have much greater motivational power than other methods [21]. The GLADIOLUS study demonstrated that one-time use of Group Behavioral Therapy (GBT) is moderately effective and cost-saving for reducing frequency, severity, and improved quality of life [34]. The competency-based approach and case methods approaches have been demonstrated as an effective strategy in modifying attitudes, knowledge, and skills that are generally more effective than traditional, passive modes of teaching. Competency refers to how one integrates the complexities of attitudes, knowledge, and skills that can be mobilized appropriately in response to specific professional situations. Nurses can provide care using action skills that require the integration and application of newly acquired knowledge. The components are to first assess the patient and then develop a plan for the patients with appropriate follow-up plans [19]. The case method approach was chosen for the educational program because it could be used with the competency-based approach as it fosters the integration of knowledge. There is a confidence that when presented with the real details, learners could analyze situations and better link theory and practice with the realities of the profession. This method uses all the resources within the competence-based approach including attitudes, knowledge, and skills. When compared with traditional lecture learners, the case method learners demonstrated high-level skill sets and were better able to analyze and problem-solve [21]. 

Lecture-style learning should only be used to encourage discussion and provide diagrams to aid understanding. To maintain interest, information is to be presented in 10- to 15-minute segments. This approach considers research that indicates that when teaching new concepts, “less is more”; attention spans wane after about 25 minutes and most people can process only about four ideas at one time [21]. 

Based on the literature, the previously discussed steps along with competency-based approaches appear promising. It is important to note that part of the success of the Kincade study was due to their educational program being supplemented by onsite consultation and guidance in dealing with specific resident situations; in addition, an interdisciplinary team approach was required to fully address the needs of the residents. Both variables were considered in turn [33]. 

The Usefulness of a Collaborative Approach

Palmer supports the idea that UI in older adults is complex in nature and demands an interdisciplinary approach to treatment [35]. In addition, the author asserts treatment requires the knowledge and specialized skills of several disciplines to achieve maximal patient outcomes. A LTC setting may foster interdisciplinary care of incontinence for older adults through the creation of teams. These include but are not limited to nursing, physical therapy, nutrition therapy, administration, and medicine. Here each discipline would bring its own expertise and skills to the table. The author devised a model of continence promotion, entitled “Prevention Focused Intervention: UI Based on the Health Promotion and Disease Prevention Model.” This model demonstrates that the concerted effort and skills of an interdisciplinary group are essential to achieving continence promotion. The model identifies UI as a public health issue, removing incontinence from a specific discipline. The public health perspective provides a broader view of UI, incorporating continence promotion as well as prevention strategies [16]. 

 In a pilot study, Karon demonstrated that there were statistically significant reductions in episodes of UI when a team approach to bladder control problems was employed [36]. In addition, this pilot study results appeared to validate and provide evidence that a bladder education program as an intervention is a valuable strategy to reduce the impact of this most debilitating problem. Researchers paired a clinical nurse specialist (CNS) with a physiotherapist to develop a team approach to continence management. Fifty adults (34 women/16 men) aged 27-90 years (mean age 68.3 years) were enrolled in a three-month bladder retraining program. Bladder diaries were used to assess the outcomes of clinical interventions. The results of the study indicated that with regards to mean episodes of UI, there was a significant difference between baseline and completion of the training program. 

The CNS completed assessments, performed post-void residual (PVR) scans, and participating in education. The physiotherapist was involved in evaluating, diagnosing, and treating patients using physical means. The work began with an assessment of the patient's condition, including a medical history review and a physical examination. Both elements are important to successfully examine and treat the patient. Though the team had no physician, they had access to one if needed for consultation [36].

Advance Practice Nurse as Team Member

 Although the nurses have specialized in the management of incontinence for more than 30 years their role has been underutilized due to lack of awareness [37]. Klay and Marfyak performed a study that utilized a continence nurse specialist in an extended care facility to design a continence program. The aim of the study was to determine if the number of incontinence episodes for an elderly female population could be decreased through an individualized continence program. Forty-two female residents who resided in nursing homes who were incontinent of urine or had urgency related to overactive bladder were included in the incontinence program. The total number of incontinent episodes for each participant was recorded one week prior to the study. Then an individualized plan of care for each patient was developed by the continence specialist and the plan of care was implemented for at least one year [6]. After this continence specialist program was implemented for one year, the number of incontinence related issues were substantially decreased. UTIs dropped from 31 to 6, pressure ulcers decreased from 15 to 2, additionally falls decreased from 18 to 7 postintervention. It is possible that reliability and validity may be skewed because all information obtained was from medical records and was dependent on the accuracy of that documentation. The sample size also limits the generalizability of the study. However, the conclusion of this study demonstrated that a continence specialist will help in LTC facilities, will improve patient outcomes, and will reduce costs of care. The continence specialist was a contracted, advanced practice registered nurse who provided expertise in assessment and pharmacology, which was consistent with her role within this study. The findings were shared with the medical staff. The specialist provided extensive in-servicing with staff and families on topics related to incontinence in seniors [6]. 

In another study aimed at advancing quality of UI are, proposed a model that utilizes NPs as consultants to nursing homes to perform the basic incontinence evaluation and treatment. A quasi-experimental research design with pre- and post-comparison data was selected utilizing four control and four experimental NP caseloads followed for 16 weeks post-onset. Findings are not yet available from this study, which is ongoing. The author proposed a new model by drawing on a successful model of psychiatric mental health nurse consultants used by nursing homes to facilitate the evaluation of behavioral aspects of dementia under a Medicare benefit. This model would allow NP nursing home consultants to perform UI evaluation and treatment in collaboration with attending physicians. Should the model prove effective, hopes are that it would obtain Medicare support. What is yet to be proven is whether a Medicare supported UI NP consultant can decrease the cost to Medicare as well as to nursing homes [9].

## Conclusions

Urinary incontinence is a major challenge in nursing homes, for both the facility staff and the residents. Even with the high prevalence of UI in the elderly population, and despite a growing body of information available for UI, professional education on UI remains a small or nonexistent part of the basic training of many health professionals. In addition, there is sparse literature available from the last decade to study and address this highly prevalent yet preventable cause of geriatric patient morbidity and mortality.

The important aspects of incontinence care in nursing homes include assessing the problem and developing a multidisciplinary team approach to its management. Nursing education is very critical, although not a simple intervention-outcome approach, it does have the capability of affecting current and future residents. Educating nurses on current evidence-based practice (EBP) guidelines and best practice recommendations for the evaluation and management of UI is needed to attain and/or maintain proficient clinical skills and to maintain competency.
